# Negative prognostic behaviour of PD-L1 expression in tongue and larynx squamous cell carcinoma and its significant predictive power in combination with PD-1 expression on TILs

**DOI:** 10.1186/s12865-024-00597-0

**Published:** 2024-01-16

**Authors:** Simin Ahmadvand, Lotf-Ali Norouzi, Yousef Mohammadi, Akbar Safaei, Bijan Khademi, Maziar Motiee-Langroudi, Abbas Ghaderi

**Affiliations:** 1 Department of Biochemistry, Microbiology, and Immunology, Faculty of Medicine, University of Ottawa, Ottawa, ON Canada; 2 Otolaryngology Research Centre, Department of Otorhinolaryngology, Shiraz University of Medical Sciences, Shiraz, Iran; 3 Shiraz Institute for Cancer Research, Faculty of Medicine, Shiraz University of Medical Sciences, Shiraz, Iran; 4 Department of Pathology, School of Medicine, Shiraz University of Medical Sciences, Shiraz, Iran; 5 Otorhinolaryngology Research Centre, Tehran University of Medical Sciences, Tehran, Iran; 6 Department of Immunology, School of Medicine, Shiraz University of Medical Sciences, Shiraz, Iran

**Keywords:** Head and Neck Squamous Cell Carcinoma, Checkpoint Inhibitor Immunotherapy, PD-1, PD-L1, Tumor Infiltrating Lymphocytes, Tumor Microenvironment, Prognostic Marker

## Abstract

**Background:**

Biomarkers that can predict outcome will improve the efficacy of treatment for HNSCC patients. In this regard, we retrospectively evaluated the prognostic effect of PD1, PD-L1, and CD45RO in tongue and larynx squamous cell carcinomas.

**Methods:**

FFPE tissue blocks of 63 larynx and 40 tongue squamous cell carcinoma samples were selected, cut into 3 µm sections, and immunohistochemically stained for PD1, PD-L1, and CD45RO. The slides were evaluated by an expert pathologist, and results were analysed using Chi-square, univariate, and multivariable Cox regression methods.

**Results:**

TC-PD-L1 expression (*P* = *0.001*) and its expression intensity (*P* = *0.002*) were significantly correlated with a higher percentage of PD-1 + tumor infiltrating lymphocytes. In univariate survival analysis, TC-PD-L1 and its expression intensity had a significant impact on both DFS (HR: 0.203; *P* = *0.003* and HR: 0.320; *P* = *0.005*) and OS (HR: 0.147; *P* = *0.002* and HR: 0.322; *P* = *0.005).* Based on the multivariate analysis, PD1 (DFS: HR: 3.202; *P* = *0.011*, OS: HR: 2.671; *P* = *0.027*) and TC-PD-L1 (DFS: HR: 0.174; *P* = *0.006*, OS: HR: 0.189; *P* = *0.009*) were found to be independent prognostic markers. In the second part, scoring systems were defined based on the expression status of PD1 and PD-L1. Patients with higher scores were expected to have longer DFS and OS. In multivariate analysis, the PD1/TC-PD-L1 (DFS: *P* = *0.001*, OS: *P* = *0.003*) scoring systems showed superior prognostic effects. Interestingly, at the highest levels of this score, none of the patients experienced recurrence or cancer-caused death.

**Conclusion:**

Collectively, this study suggests negative prognostic behaviour for TC-PD-L1 protein and introduces the PD-1/TC-PD-L1 scoring system as a strong prognostic marker in OS and DFS prediction of tongue and larynx HNSCC patients.

**Supplementary Information:**

The online version contains supplementary material available at 10.1186/s12865-024-00597-0.

## Background

Head and neck cancer is one of the most common cancers around the world and accounts for more than 330,000 deaths annually [1]. Head and neck squamous cell carcinoma (HNSCC) represents about 90% of all head and neck cancers [2].

Tumor infiltrating lymphocytes (TILs) are shown to have prognostic significance in cancer patients [3, 4]. Among different immune cell types, high infiltration of cytotoxic (CD8 +) and memory T cells (CD45RO +) is strongly associated with longer disease-free survival (DFS) and/or improved overall survival (OS) in different cancer types [5–8]. The cytotoxic potential of CD8 + T cells and the ability of CD45RO + memory cells to recall previously encountered antigens may be the cause of the positive prognostic role of these cells. However, this is a two-way interaction, and tumor-induced immune inhibitory mechanisms have the potential to interrupt the anti-tumour activity of the effector immune cells [9]. One such inhibitory effect occurs through the expression of PD-L1 on tumor cells, which binds to its receptor, PD-1, on activated T cells and inhibits their anti-tumor activity. In a normal immune response, after TCR activation, effector/memory T cells express elevated levels of the inhibitory receptor PD-1, which, upon binding to its ligands PD-L1 or PD-L2, prevents T-cell over-reactivity and limits self-tissue damage. The expression pattern of these two ligands is different. While PD-L2 expression is mainly restricted to activated dendritic cells and myeloid cells, PD-L1 expression is found in a wide range of cell types, including activated T lymphocytes and tumor cells [10]. Clinical trials have shown that the systemic administration of anti-PD-1 or anti-PD-L1 blocking antibodies in recurrent or metastatic cancer patients improves their prognosis [11]. Topalian et al. in their study injected anti-PD-1 antibodies into 296 patients with advanced melanoma, non-small cell lung cancer, colorectal, renal, and prostate cancers. They observed complete or partial responses in 18% of non-small-cell lung cancer patients, 28% of melanoma patients, and 27% of renal-cell cancer patients. To evaluate the association between PD-L1 expression and response to therapy, pre-treatment tumor specimens from 42 patients were immunohistochemically stained for PD-L1 expression 36% of PD-L1-positive patients and none of the PD-L1-negative patients were responders [12]. These results show the beneficial effect of PD-1/PD-L1 blockade in recurrent or metastatic tumors, especially PD-L1-positive ones. However, about 40%–45% of patients fail to respond to checkpoint inhibitor therapy [13, 14]. Finding biomarkers that can best predict outcome is important to improving treatment efficacy. Evaluation of the inhibitory markers PD-1 and PD-L1 in combination with TIL subsets may help achieve this goal. As we had previously observed the positive prognostic effect of CD45RO + TILs in breast cancer [15], in the current study the expression pattern of PD-1, PD-L1, and CD45RO + immune cells and their relevance to clinicopathological features of disease were evaluated by immunohistochemical staining of the HNSCC tumor tissues. To minimize the confounding effects of tumor heterogeneity, we just focused on tongue and larynx SCCs as the most common subtypes.

## Results

### Expression analysis

#### Clinicopathologic characteristics of the study population

A population of 103 patients with primary tongue (38.8%) and larynx (61.2%) SCC were enrolled in the study. Most of the patients were male (72.8%). The mean age of patients at the time of operation was 59.17 (27–87) years. The mean of patients’ DFS and OS were 37.2 (0.8–79.8) and 40.0 (3.1–79.8) months, respectively. During follow-up time, 29 (28.2%) cases experienced disease recurrence, and 30 (29.1%) cases expired due to cancer. Sixty-three percent of patients were in TNM-stage III, and 35% of patients were positive for lymph node metastases. More details about the study population have been summarized in Supplementary Table 1.

#### PD-L1 expression by tumor cells is associated with PD-1 expression by TILs

**Fig. 1:**
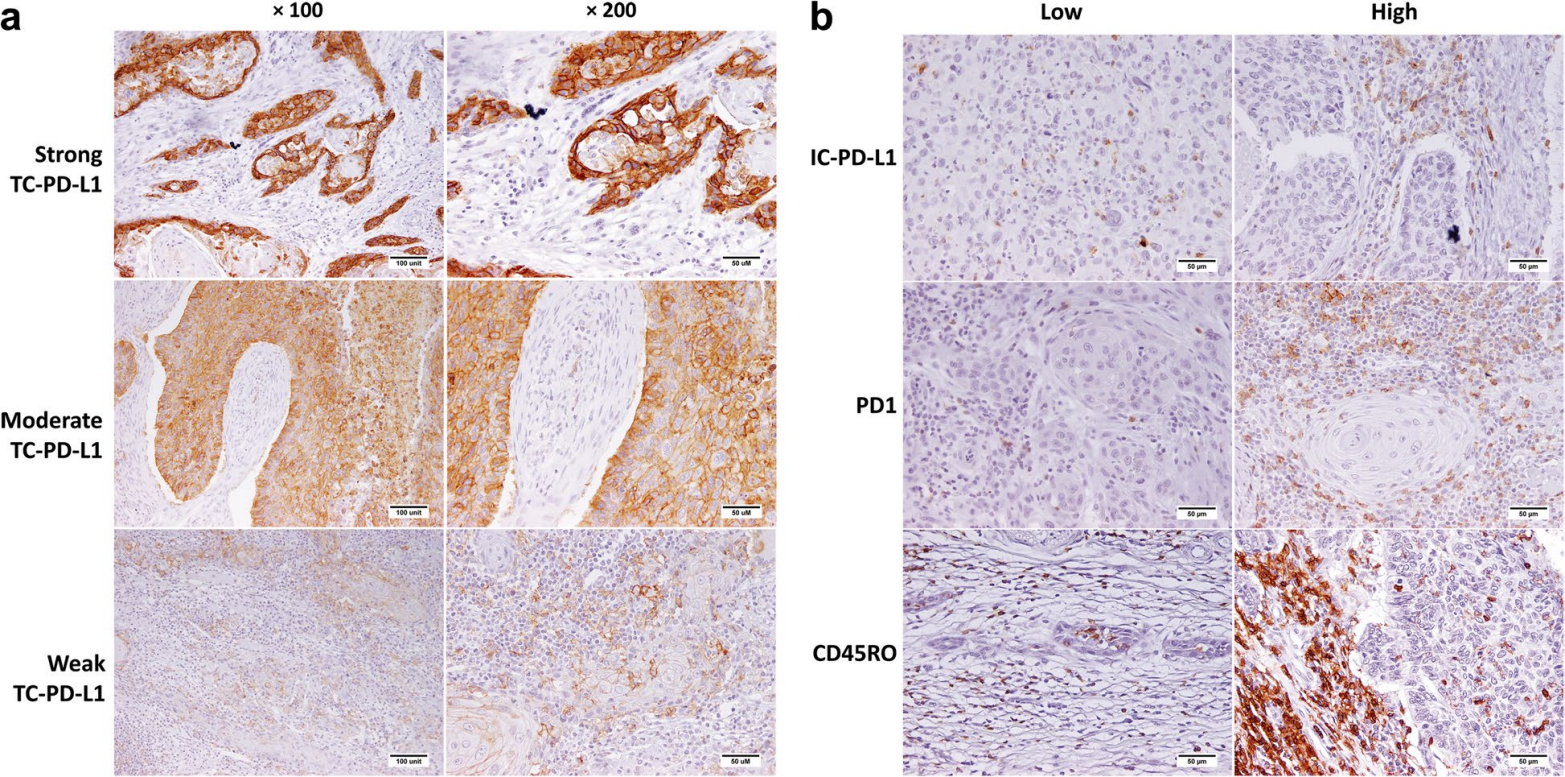
Immunohistochemical staining of tongue and larynx squamous cell carcinoma FFPE tissue samples for PD-1, PD-L1, and CD45RO markers.** A** Shows different expression intensities of PD-L1 by tumor cells. **B** Represents examples of low and high expression of PD-1 and PD-L1 by immune cells, as well as low and high infiltration of CD45RO + immune cells. IC-PD-L1: PD-L1 expression by immune cells, TILs: tumor infiltrating lymphocytes

PD-1, PD-L1, and CD45RO expressions were detected by IHC staining (Fig. 1), and the results were evaluated by an expert pathologist. We did not have the PD1 staining data for eight samples. However, about half (51.6%) of the remaining ones were highly positive for PD-1. While PD-1 expression was restricted to TILs, PD-L1 was positive in both tumor and immune cells.

According to the results, TC-PD-L1 was positive in 61.2% of tumors. A high IC-PD-L1 was detectable in 60.2% of samples (IC-PD-L1 > 5%). With regard to PD-L1 expression intensity, 27%, 33.3%, and 39.7% of positive tumor cells showed weak, moderate, and strong expression intensities, respectively. In 52.4% of samples, CD45RO + TILs comprised over 60% of total lymphocytic infiltrates.

We also studied if TC-PD-L1 expression is associated with other studied markers. The results showed that both TC-PD-L1 positive expression (*P* = 0.001) and expression intensity (*P* = 0.002) were associated with high PD-1 expressing TILs. TC-PD-L1 did not show a significant association with IC-PD-L1 (*P* = 0.391) or CD45RO + TILs (P = 0.229). High CD45RO + immune cells (*P* = 0.001) and high IC-PD-L1 (*P* 0.001) were also associated with high PD-1 expression (Table 1).

**Table 1:** Association of PD1, PD-L1 and CD45RO + TILs with clinicopathological features of the disease

Variable	PD-1		CD45RO		TC-PD-L1		IC-PD-L1	
	** < 10% (%)**	** > 10% (%)**	** < 60%**	** > 60%**	** < 1%**	** > 1%**	** < 5%**	** > 5%**
**Age**	0.220		0.618		0.124		0.161	
< 58	26 (27.4)	21 (22.1)	26 (25.2)	26 (25.2)	24 (23.3)	28 (27.2)	17 (16.5)	35 (34.0)
> 58	20 (21.1)	28 (29.5)	23 (22.3)	28 (27.3)	16 (15.5)	35 (34.0)	24 (23.3)	27 (26.2)
**Sex**	0.606		0.103		0.395		0.657	
Male	35 (36.8)	35 (36.8)	32 (31.1)	43 (41.7)	31 (30.1)	44 (42.7)	31 (30.1)	44 (42.7)
Female	11 (11.6)	14 (14.7)	17 (16.5)	11 (10.7)	9 (8.8)	19 (18.4)	10 (9.7)	18 (17.5)
**Grade**	0.056		0.303		**0.026**		0.476	
I/II ii	31 (34.8)	42 (47.2)	9 (9.3)	9 (9.3)	26 (26.8)	53 (54.6)	28 (28.9)	51 (52.6)
III	11 (12.4)	5 (5.6)	35 (36.1)	44 (45.4)	11 (11.4)	7 (7.2)	8 (8.2)	10 (10.3)
**T stage**	0.073		0.939		**0.022**		0.211	
T1/T2	10 (10.9)	20 (21.7)	15 (15.0)	18 (18.0)	7 (7.0)	26 (26.0)	10 (10.0)	23 (23.0)
T3/T4	33 (35.9)	29 (31.5)	31 (31.0)	36 (36.0)	30 (30.0)	37 (37.0)	29 (29.0)	38 (38)
**Lymph node**	0.419		0.177		0.776		0.862	
Involved	11 (12.1)	16 (17.6)	18 (18.2)	14 (14.1)	11 (11.1)	21 (21.2)	13 (13.1)	19 (19.2)
Free	32 (35.2)	32 (35.2)	28 (28.3)	39 (39.4)	25 (25.3)	42 (42.4)	26 (26.3)	41 (41.4)
**TNM stage**	0.144		0.880		0.089		0.177	
I + II	9 (9.8)	17 (18.5)	13 (13.0)	16 (16.0)	7 (7.0)	22 (22.0)	8 (8.0)	21 (21.0)
III + IV	34 (37.0)	32 (34.8)	33 (33.0)	38 (38.0)	30 (30.0)	41 (41.0)	31 (31.0)	40 (40.0)
**Recurrence**	0.177		0.065		**0.001**		**0.046**	
Yes	15 (15.8)	10 (10.5)	18 (17.5)	11 (10.7)	4 (3.9)	25 (24.3)	16 (15.6)	13 (12.6)
No	31 (32.6)	39 (41.1)	31 (30.1)	43 (41.7)	36 (35.0)	38 (36.8)	25 (24.3)	49 (47.5)
**Cancer death**	0.516		0.105		**0.000**		0.175	
Yes	14 (14.7)	12 (12.6)	18 (17.5)	12 (11.7)	3 (2.9)	27 (26.2)	15 (14.6)	15 (14.6)
No	32 (33.7)	37 (38.9)	31 (30.1)	42 (40.8)	37 (35.9)	36 (35.0)	26 (25.2)	47 (45.6)
**Smoking**	0.621		0.272		**0.033**		0.832	
Yes	25 (27.8)	31 (34.4)	26 (26.5)	35 (35.7)	28 (28.6)	33 (33.7)	23 (23.5)	38 (38.8)
No	17 (18.9)	17 (18.9)	10 (20.4)	17 (17.3)	9 (9.2)	28 (28.6)	15 (15.3)	22 (22.4)
**PD1**			**0.001**		**0.001**		**0.000**	
< 10%			29 (30.5)	17 (17.9)	26 (27.4)	20 (21.1)	27 (28.4)	19 (20.0)
> 10%			14 (14.7)	35 (36.8)	12 (12.6)	37 (38.9)	11 (11.6)	38 (40.0)
**CD45RO**	**0.001**				0.229		0.070	
< 60%	29 (30.5)	14 (14.7)			22 (21.4)	27 (26.2)	24 (23.3)	25 (24.3)
> 60%	17 (17.9)	35 (36.8)			18 (17.5)	36 (35.0)	17 (16.5)	37 (35.9)
**TC-PD-L1**	**0.001**		0.229				0.391	
< 1%	26 (27.4)	20 (21.1)	22 (21.4)	18 (17.5)			18 (17.5)	22 (21.4)
> 1%	20 (21.1)	37 (38.9)	27 (26.2)	36 (35.0)			23 (22.3)	40 (38.8)
**IC-PD-L1**	**0.000**		0.070		0.391			
< 5%	27 (28.4)	11 (11.6)	24 (58.5)	17 (16.5)	18 (17.5)	23 (22.3)		
> 5%	19 (20.0)	38 (40.0)	25 (24.3)	37 (35.9)	22 (21.4)	40 (38.8)		

*TC-PD-L1* PD-L1 expression by tumor cells, *IC-PD-L1* PD-L1 expression by immune cell

#### Association of PD-1, PD-L1, and CD45RO + TILs with HNSCC clinicopathologic features

TC-PD-L1 was significantly correlated with tumor grade (*P* = 0.026) and T-stage (*P* = 0.022). Most of the patients who had experienced recurrence and cancer related deaths were in the TC-PD-L1 positive group (*P* = 0.001 and *P* < 0.001, respectively). Negative TC-PD-L1 status was significantly correlated with smoking (*P* = 0.033).

Among all clinicopathological features, IC-PD-L1 showed a significant correlation with disease recurrence (*P* = 0.046). Contrary to TC-PD-L1, most of the recurrence-free patients were in the high IC-PD-L1 expressing group.

Chi-square analyses showed no significant differences between patient groups based on PD-1 and CD45RO + TILs expression. CD45RO + TILs and PD-1 expression showed a near-significant association with recurrence (*P* = 0.065) and tumor grade (*P* = 0.056), respectively (Table 1).

### Survival analysis

#### PD-1 and TC-PD-L1 expression have independent prognostic effects on DFS and OS

In survival analysis, Cox regression and Kaplan–Meier methods were used to study the prognostic impact of PD-1, PD-L1, and CD45RO + TILs on DFS and OS.

In univariate Cox regression analyses, negative TC-PD-L1 expression was associated with longer DFS (HR: 0.203; 95% CI: 0.070–0.583). TC-PD-L1 expression intensity also had a prognostic effect on DFS, so that patients with negative and weak (0/1 +) expression intensities had a better DFS than those with moderate and strong (2 + /3 +) expression intensities (HR: 0.320; 95% CI: 0.145–0.704). In contrast, patients with weak IC-PD-L1 intensity were more prone to recurrence, with an HR of 2.378 (95% CI: 1.078–5.247). Although *P*-values did not reach statistical significance, low IC-PDL-1 and CD45RO infiltration showed a trend of negative effect on DFS with HRs of 2.015 and 1.902 (95% CI: 0.969–4.191 and 0.898–4.029). Among the studied markers, only TC-PD-L1 protein expression (HR: 0.147; 95% CI: 0.045–0.485) and expression intensity (HR: 0.322; 95% CI: 0.147–0.704) could predict patients’ OS.

All the clinicopathologic parameters, including age, sex, histological grade, lymph node metastases, and TNM stage, showed an insignificant effect on both DFS and OS (Table 2).

**Table 2:** Survival analysis for PD1, PD-L1, CD45RO + TILs and clinicopathological features

**Variable**		**DFS**					**OS**				
		**β**	**S.E**	**HR**	**95% CI for HR**	***P*-value**	**β**	**S.E**	**HR**	**95% CI for HR**	***P*-value**
**Univariate Cox regression**											
**Age (Y)**	** ≤ 58**			1(Ref)					1 (Ref)		
	** > 58**	0.067	0.372	1.070	0.516–2.217	0.856	0.122	0.366	1.130	0.551–2.316	0.739
**Sex**	**Male**			1 (Ref)					1 (Ref)		
	**Female**	0.312	0.392	1.365	0.634–2.942	0.426	-0.031	0.408	0.970	0.436–2.157	0.940
**Origin**	**Tongue**			1(Ref)					1 (Ref)		
	**Larynx**	-0.568	0.372	0.567	0.273–1.176	0.127	-0.342	0.377	0.711	0.339–1.489	0.365
**Histological Grade**	**I/II**			1(Ref)					1(Ref)		
	**III**	0.483	0.467	1.621	0.649–4.047	0.301	0.573	0.473	1.774	0.702–4.484	0.226
**T stage**	**T3/T4**			1(Ref)					1 (Ref)		
	**T1/T2**	-0.101	0.405	0.904	0.409–1.999	0.803	-0.284	0.402	0.753	0.342–1.657	0.481
**Lymph node status**	**Free**			1 (Ref)					1 (Ref)		
	**Involved**	0.541	0.383	1.717	0.811–3.635	0.157	0.415	0.380	1.515	0.720–3.189	0.274
**PD1**	** > 10%**			1 (Ref)					1 (Ref)		
	** < 10%**	0.537	0.409	1.711	0.768–3.812	0.189	0.294	0.404	1.342	0.608–2.962	0.466
**TC-PD-L1**	** > 1%**			1(Ref)					1 (Ref)		
	** < 1%**	-1.596	0.540	0.203	0.070–0.583	**0.003**	-1.918	0.609	0.147	0.045–0.485	**0.002**
**IC-PD-L1**	** > 5%**			1 (Ref)					1 (Ref)		
	** < 5%**	0.701	3.74	2.015	0.969–4.191	**0.061**	0.558	0.372	1.747	0.842–3.625	0.134
**TC-PDL1 intensity**	**2 + /3 + **			1 (Ref)					1 (Ref)		
	**0/1 + **	-1.140	0.403	0.320	0.145–0.704	**0.005**	-1.134	0.399	0.322	0.147–0.704	**0.005**
**IC-PD-L1 intensity**	**2 + /3 + **			1 (Ref)					1 (Ref)		
	**1 + **	0.866	0.404	2.378	1.078–5.247	**0.032**	0.639	0.418	1.894	0.835–4.299	0.127
**CD45RO**	** > 60%**			1 (Ref)					1 (Ref)		
	** < 60%**	0.643	0.383	1.902	0.898–4.029	**0.093**	0.531	0.374	1.700	0.818–3.536	0.155
**Smoking**	**Yes**			1 (Ref)					1 (Ref)		
	**No**	0.699	0.379	2.013	0.957–4.233	**0.065**	0.369	0.375	1.446	0.694–3.013	0.325
**TNM-stage**	**III/IV**			1 (Ref)					1 (Ref)		
	**I/II**	-0.341	0.437	0.711	0.302–1.674	0.435	-0.549	0.437	0.578	0.245–1.361	0.210
**Multivariate Cox regression**											
**PD1**	** > 10%**			1 (Ref)					1 (Ref)		
	** < 10%**	1.164	0.458	3.202	1.305–7.860	**0.011**	0.982	0.444	2.671	1.120–6.370	**0.027**
**TC-PD-L1**	** > 1%**			1(Ref)					1(Ref)		
	** < 1%**	-1.747	0.635	0.174	0.050–0.605	**0.006**	-1.665	0.634	0.189	0.055–0.655	**0.009**

*DFS* disease-free survival, *OS* overall survival, *S.E* standard error, *HR* hazard ratio, *95% CI for HR 95%* confidence interval for hazard ratio, *TC-PD-L1* PD-L1 expression by tumor cells, *IC-PD-L1* PD-L1 expression by immune cells

To investigate the independent prognostic effect of the markers, all variables in the univariate Cox regression with a *P*-value ≤ 0.2 were entered into a multivariate Cox regression model and adjusted for age, sex, tumor origin, tumor grade, lymph node metastasis, TNM stage, and smoking behaviour. Based on the results, TC-PD-L1 (negative vs. positive, HR: 0.174; 95%CI: 0.050–0.605 and HR: 0.189; 95%CI: 0.055–0.655) and PD-1 (low vs. high, HR: 3.202; 95%CI: 1.305–7.860 and HR: 2.671; 95%CI: 1.120–6.370) were the two variables that predicted both DFS and OS independently (Table 2).

#### Collective effect of PD-1 and TC-PD-L1 on HNSCC prognosis

In the above analyses, high PD-1 and negative TC-PD-L1 showed positive prognostic effects on disease outcome as independent prognostic markers. In this regard, we defined a scoring system based on the combination of these parameters and studied their prognostic significance as a score. For this purpose, each of the above-mentioned parameters was assigned a number 1 in favour of the patient, and their corresponding opposite groups were given a number 0, and the patient’s score was calculated by summing up these numbers. In our scoring systems, we hypothesised that both DFS and OS would improve from score 0 (S0) to the highest possible score (S2). In more detail, S2 patients were expected to benefit from longer DFS and OS compared to S1 and S0 groups, respectively.

**Fig. 2:**
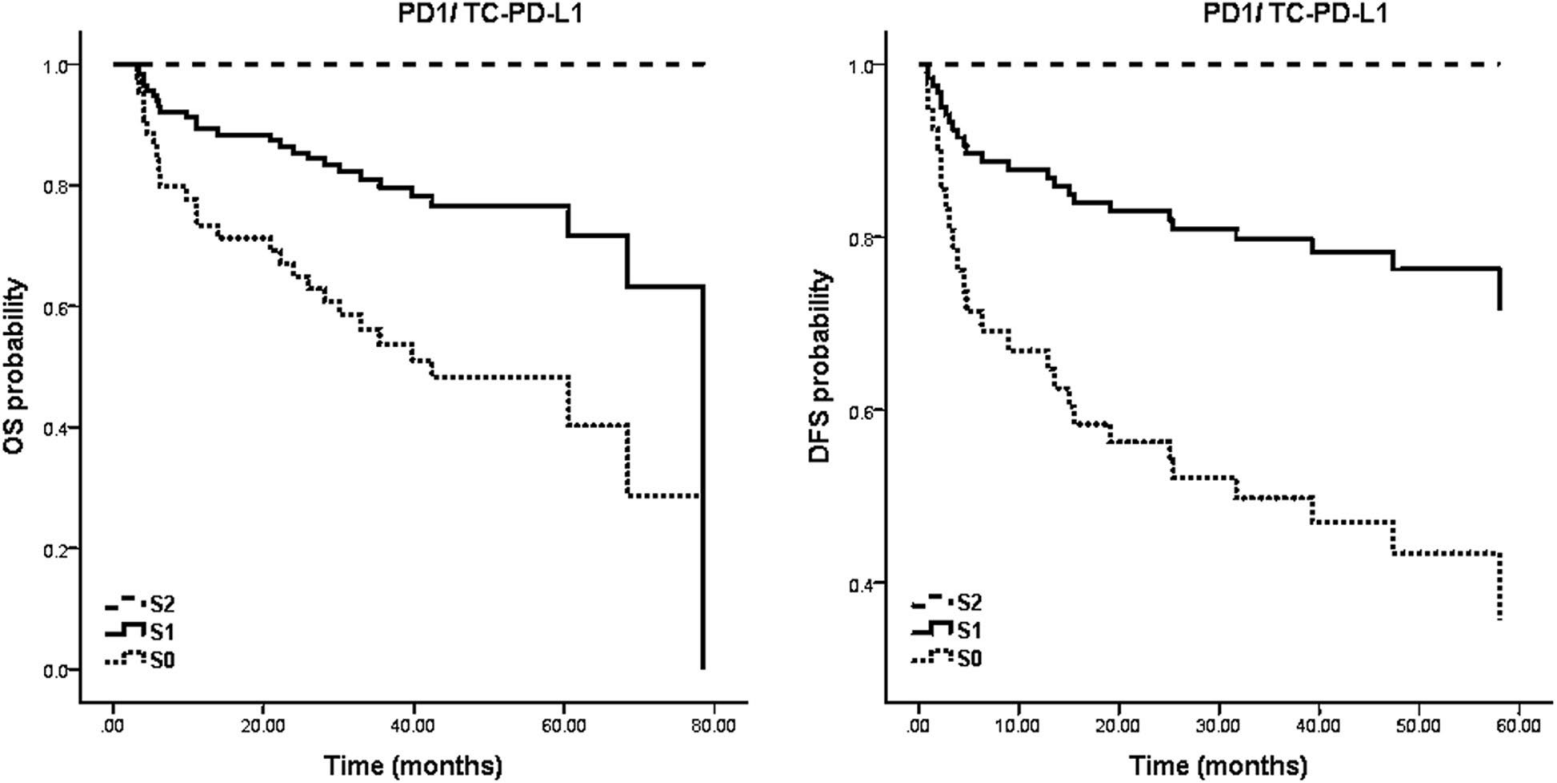
Kaplan–Meier (K-M) curves plotted for OS and DFS probability to demonstrate the prognostic power of the PD1-TC-PD-L1 scoring system. As it is interpreted from the figures, the scores have significant prognostic potential, and patients with higher scores have benefited from longer DFS and OS. K-M curves show that in the patients with the highest score of PD-1/TC-PD-L1, none of the patients have experienced disease recurrence or death. OS: overall survival, DFS: disease-free survival, TC-PD-L1: PD-L1 expression by tumor cells

As the K-M curves in (Fig. 2) represent, PD1-TC-PD-L1 was a significant OS (0.046) and DFS (*P* = 0.021) predictor in our study population in such a way that none of the patients with an S2 score (*n* = 12, 12.6%) experienced recurrence or cancer-caused death during the follow-up time. For multivariate survival analyses, we combined these event-free score levels with their previous score levels to be able to build a survival model.

**Fig. 3:**
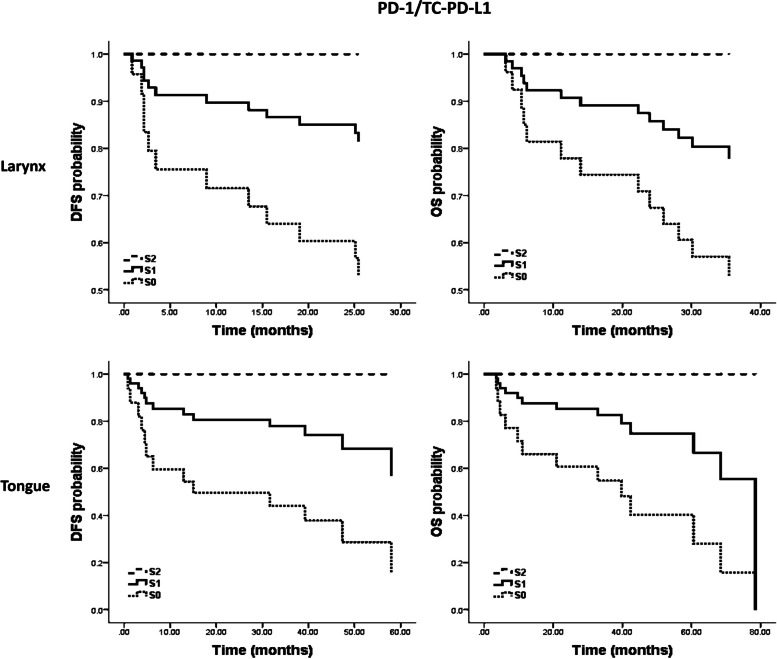
Kaplan–Meier (K-M) curves are plotted for the PD-1/TC-PD-L1 scoring system in the tongue and larynx SCC separately. As the graphs show, the PD-1/TC-PD-L1 scoring system separates patient groups and has the potential for OS and DFS prediction in tongue and larynx squamous cell carcinomas separately. Patients within the highest score levels showed the best prognosis, and none of them experienced recurrence or cancer-caused death

PD-1/TC-PD-L1 (High vs. low; DFS:HR: 0.282; 95% CI: 0.121–0.656; OS: HR: 0.239; 95% CI: 0.101–0.563) showed a significant prognostic effect among all the potential prognostic markers in the survival model. We also evaluated the predictive potential of these factors in tongue and larynx SCCs separately; patients were divided based on their tumor origin to check the independent prognostic power of this score in tongue and larynx SCCs separately. As the K-M curves in (Fig. 3) show, in both tumor groups, the PD1/TC-PD-L1 scoring system predicted DFS and OS, and patients within the highest score level did not experience recurrence and/or cancer-caused death.

## Discussion

Check-point inhibitor immunotherapies, especially PD-1/PD-L1 blockade, are promising therapeutic approaches in a number of cancers, including melanoma, lung cancer, and head and neck cancer. Recently, tumor infiltrating lymphocytes, particularly CD8 + and CD45RO + T cells, seem to have a positive prognostic impact on cancer outcome. In this regard, in the current study, we evaluated the expression of PD-1 and PD-L1 as well as the extent of CD45RO + immune cell infiltration in tongue and larynx SCCs and studied their prognostic effects on patients’ DFS and OS.

In our study, PD-L1 expression by tumor cells was detectable in about 60% of samples. Tumor cells from tongue SCC seemed to be more likely to express PD-L1. However, PD-1/PD-L1 expression by immune cells and CD45RO + infiltration were not statistically different among tongue and larynx SCC samples. Infiltration of CD45RO + immune cells was present in all the tissues, and their high infiltration showed a significant correlation to a high percentage of PD-1 + and PD-L1 + TILs. This result is in concert with the fact that immune activation is followed by co-inhibitory molecule expression that, as a part of the negative feedback loop, regulates immune cells over-reactivity and controls self-tissue damage [16]. PD-1 is one of the first inhibitory checkpoint proteins that is expressed on activated T cells upon TCR signalling [10] and its expression is also induced by IL-2, IL-7, IL-15, and IL-21 cytokines that are critical for T-cell activation and proliferation [17]. In an immune response that results in antigen clearance, PD-1 expression is reduced in the absence of TCR signalling. In chronic conditions like viral infections where the immune response fails to completely resolve the target antigen, PD-1 will be continuously expressed on T cells to reduce the risk of autoimmunity by ligation to PD-L1 and PD-L2 [17, 18]. In this study, it was observed that PD-L1 expression by tumor cells was significantly correlated with the presence of PD-1 + TILs. Several studies have reported that PD-L1 expression is regulated by IFN-ɣ secretion [19]. A high percentage of PD-1 + TILs may represent the formation of a robust anti-tumor immune response and IFN-ɣ secretion that induces PD-L1 expression by tumor cells. PD-L1 expressing tumors consequently suppress the effector function of cytotoxic T cells and facilitate tumor immune escape. Another possible explanation is that the anti-tumor immune response, by killing PD-L1 negative tumor cells, has selected PD-L1 expressing cells, which consequently facilitate tumor immune scape by suppressing effector immune cells.

In the current study, the prognostic significance of PD-1, PD-L1, and CD45RO + TILs was studied. Univariate Cox regression analyses showed that negative TC-PD-L1 (DFS, HR: 0.203, *P* = 0.003, OS, HR: 0.147, *P* = 0.002) and its negative or weak expression intensity (DFS: HR: 0.320, *P* = 0.005, OS: HR: 0.322, *P* = 0.005) had a positive prognostic effect on both DFS and OS. Conversely, a low percentage of PD-L1 + immune cells and its weak expression intensity showed a negative effect on DFS in such a way that the hazard of recurrence in patients with low IC-PD-L1 expression and expression intensity were 2.015 (*P* = 0.061) and 2.378 (*P* = 0.032) fold greater than their opposite corresponding groups, respectively. This opposite behaviour may suggest that while PD-L1 expression on tumor cells is representative of their potential to suppress the immune response, its expression on immune cells indicates that they have probably been activated and, as a result, are expressing PD-1/PD-L1 on their cell surface.

In multivariate analyses, PD-L1 expression by tumor cells and PD-1 expression by immune cells showed independent prognostic effects on both DFS and OS. The hazard of recurrence and death in patients with negative TC-PD-L1 was 0.174 (*P* = 0.006) and 0.189 (*P* = 0.009) fold lower than in the PD-L1 positive group, respectively. Patients with low PD-1 + TILs were more likely to experience recurrence and cancer-caused death, with HRs of 3.202 (*P* = 0.011) and 2.671 (*P* = 0.027). Consistent with our results, a research group reported positive prognostic effects for PD-1 and IC-PD-L1 in ovarian cancer. However, contrary to our results, they reported a positive prognostic effect for TC-PD-L1 [20]. In a study conducted on HPV-negative HNSCC samples, PD-1 positive TILs had a favourable effect on disease outcome [21].

Although PD-1 has been identified for its role in T cell exhaustion, based on current knowledge, it is assumed that its expression is an indicator of an Ag-specific T cell response. This is why it has been recommended to isolate tumor specific CD8 + T cells from PD-1 expressing TILs [22]. In this regard, Simon et al. [23] isolated melanoma-specific CD8 + T cells from tumor tissue of patients as well as peripheral blood from both patients and healthy donors. They found a population of tumor specific CD8 + T cells that, after TCR activation, were unable to express PD-1. It was revealed that the PD-1 promoter in these cells is constitutively hyper-methylated. Promoter methylation is one of the common epigenetic changes that silence gene expression by inhibiting the binding of transcription factors to their proper sites [24]. Interestingly, after PD-1/PD-L1 blockade, the cytotoxic capacity of PD-1 expressing CD8 + T cells was significantly higher than the cytotoxic potential of the PD-1 negative group. It may indicate the role of PD-1 in proper T cell activation. Additionally, according to literature, in a normal immune response, upon TCR activation and CD28 signalling, NFAT and AP1 are expressed in T cells and form an NFAT-AP1 complex that binds to the promoter of the PD-1 gene and leads to its demethylation and consequently PD-1 expression [25, 26]. In chronic conditions where CD28 signalling and AP1 transcription factors are inhibited, other transcription factors as well as partner-less NFAT1 cause constitutive expression of PD-1 and a hypo-responsive T cell phenotype. So, PD-1 expression alone is not an indicator of T cell exhaustion. However, due to the chronic condition of tumor tissue and the presence of different immunosuppressive factors, PD-1 + CD8 + T cells in the tumor microenvironment are expected to be in their hypo-responsive state, but they are representative of anti-tumor immune response formation. The PD-1 + exhausted TILs may have the potential to be invigorated by the PD-1/PD-L1 blockade.

Considering these findings, we introduced a scoring system based on the PD-1 and TC-PD-L1 expression status of the resected primary tumors. The PD-1/TC-PD-L1 scoring system was a superior prognostic variable in predicting patients’ DFS and OS.

Although this score is obtained through immunohistochemical evaluation of the resected primary tumors, it might provide a summarized data about the tumor immunogenicity, its inhibitory immune checkpoint status, and the epigenetic potential of tumor cells in PD-L1 expression in individual patients. For instance, S2 patients in the PD-1/TC-PD-L1 scoring system probably are epigenetically unable to express PD-L1 and have developed a proper anti-tumor immune response that gives them the chance of having a longer DFS and OS. Additionally, patients were separated based on their tumor origin, and the scores were still predictive in both tongue and larynx patients. This may indicate that the predictive potential of these markers is independent of the tumor origin.

It was expected that higher scores would benefit from a better prognosis than lower ones. It is noteworthy that none of the patients with an S2 score of PD-1/TC-PD-L1 experienced recurrence or cancer-caused death during the follow-up. It did not happen due to a shorter follow-up time because the mean follow-up time of the S2 PD-1/TC-PD-L1 patients was 51.66 months. Even after grouping patients based on their tumor origin, patients within the highest score levels did not experience recurrence and/or cancer-caused death.

According to our findings, identifying patients with elevated PD1/TC-PD-L1 expression may help prioritize them as immunotherapy treatment candidates. Patients with increased PD-1 expression in TILs may have a more potent immune response against the tumor and may be more likely to respond to anti-PD-1/PD-L1 therapies.

## Conclusion

Generally, in this study, it was observed that the prognostic behaviour of the PD-L1 protein on immune and tumor cells is different. PD-1 and PD-L1 expression by immune cells showed significant positive prognostic effects. This may suggest that regardless of the inhibitory signalling, the expression of PD-1 and PDL1 on TILs is indicative of the formation of an effective anti-tumor immune response during tumor progression. The classification system that was defined based on PD-1 expression on TILs and TC-PD-L1 significantly predicted disease outcome. We suggest repeating the same work on a larger HNSCC study population to check the reproducibility of the results.

### Patients and method

#### Study population

This study was retrospectively conducted on FFPE tissue samples from 103 patients with approved tongue and larynx SCC who had undergone surgical removal of their primary tumors between 2012 and 2017 as their first-line treatment. The samples were obtained from Khalili Hospital, affiliated with Shiraz University of Medical Sciences, Shiraz, Iran. All the patients included in this study had not received pre-surgery chemotherapy and/or radiotherapy, and their clinicopathologic and follow-up data were available. Hematoxylin and Eosin (H&E) slides of patients were evaluated by an expert pathologist to select proper tissue blocks with a dominant tumor area and TIL infiltration. The selected FFPE tissue blocks were retrieved from the hospital’s archive and prepared for immunohistochemical staining for the desired markers. The experimental protocol of this study was approved by the Ethics Committee of Shiraz University of Medical Sciences [ethic code: IR.SUMS.REC.1398.251].

#### Immunohistochemistry (IHC)

Selected tissue blocks were cut into 3µm sections and mounted on positively charged IHC slides. Slides were heated to 61ºc and deparaffinized in xylene for 30 min. Masked antigens were retrieved in Tris–EDTA (pH 9) by the heat-induced epitope retrieval method in a pressure cooker for about 20 min. Slides were cooled down in cold water and washed twice in 1 × PBS for 5 min each. After that, endogenous peroxidases and non-hydrophobic protein interactions were blocked by applying 10% H2O2 and 10% goat serum, respectively. Anti-PD-1 (mouse monoclonal antibody, clone IHC001, 1/100, GenomeMe, Richmond, Canada), anti-PD-L1 (rabbit monoclonal antibody, clone IHC411, 1/100, GenomeMe, Richmond, Canada), and anti-CD45RO (mouse monoclonal antibody, Clone UCHL, 1/300, Dako, Denmark) primary antibodies were added and incubated for 1 h in a humid chamber. After adequate washing, visualisation was conducted by the Master Polymer Plus Detection System [Master Diagnostica, Granada, Spain] according to the manufacturer’s recommendations. Finally, slides were counterstained with haematoxylin and permanently mounted with mounting medium (Entellan, Merck, Darmstadt, Germany). Human tonsils were used as positive control tissue. Additionally, as a negative control, primary antibodies were replaced by PBS to ensure the proper function of the detection kit.

#### IHC interpretation

Immunohistochemically stained tissue sections were evaluated by an expert pathologist who was blinded to patients’ clinicopathologic and follow-up data. Necrotic areas and regions with any other artifacts were not considered for positive area evaluation.

For PD-1, results were reported as 0–5%, 5–10%, and > 10% of TILs positive for PD-1. PD-L1 expression was reported as < 1%, 1–5%, and > 5% based on the percent of positive cytoplasmic and/or membranous expression of PD-L1 by tumor cells (TC-PD-L1) and immune cells (IC-PD-L1) separately. PD-L1 expression intensity was also reported as weak, moderate, and strong based on the DAB intensity. CD45RO + immune cells were reported as 0–30%, 30–60%, and > 60% of immune cells infiltrating the tumor microenvironment. However, in the analyses, TC-PD-L1 was dichotomized into negative and positive expression for below and above the 1% cut-off point. PD-1, IC-PD-L1, and CD45RO were categorised into low and high groups based on the 10%, 5%, and 60% cut-off points, respectively. Twenty samples were selected randomly and re-evaluated by the pathologist (blindly) to check the reproducibility and reliability of the results.

### Statistical analysis

Statistical analyses were conducted by SPSS, version 20. Chi-square test was employed to study the association of the studied markers with clinicopathologic disease features. Survival times were defined as disease-free survival (DFS), the interval between operation and recurrence or last follow-up, and overall survival (OS), the period between operation and cancer-caused death or last follow-up. Univariate and multivariable Cox regression tests were conducted to study the prognostic effect of the markers on patients’ DFS and OS. In multivariable analysis, the backward elimination method was employed. Survival curves were plotted by the Kaplan–Meier method. *P*-values less than 0.05 were considered significant.

### Supplementary Information

**Additional file1: Supplementary Table 1.** Clinicopathological characteristic of the study population.

## Data Availability

The data set used or analysed during this study are available from the corresponding author on reasonable request.
