# Supplementary material for: Negative prognostic behaviour of PD-L1 expression in tongue and larynx squamous cell carcinoma and its significant predictive power in combination with PD-1 expression on TILs
Source: BMC Immunol. 2024 Jan 16;25:7. doi: 10.1186/s12865-024-00597-0 (PMC10790382; doi:10.1186/s12865-024-00597-0)
Supplement: Supplementary file 1 — Additional file1: Supplementary Table 1. Clinicopathological characteristic of the study population. [file 12865_2024_597_MOESM1_ESM.docx]

| **Characteristics** | | **N (%)** |
| --- | --- | --- |
| **Sex** | **Male** | 75 (72.8) |
|  | **Female** | 28 (27.2) |
| **Origin** | **Larynx** | 63 (61.2) |
|  | **Tongue** | 40 (38.8) |
| **Recurrence** | **Yes** | 29 (28.2) |
|  | **No** | 74 (71.8) |
| **Cancer death** | **Yes** | 30 (29.1) |
|  | **No** | 73 (70.9) |
| **Chemotherapy** | **Yes** | 67 (67.7) |
|  | **No** | 32 (32.3) |
|  | **Unknown** | 4 |
| **Radiotherapy** | **Yes** | 87 (87.9) |
|  | **No** | 12 (12.1) |
|  | **Unknown** | 4 |
| **Smoking** | **Yes** | 61 (62.2) |
|  | **No** | 37 (37.8) |
|  | **Unknown** | 5 |
| **T-stage** | **T1** | 7 (7.0) |
|  | **T2** | 26 (26.0) |
|  | **T3** | 62 (62.0) |
|  | **T4** | 5 (5.0) |
|  | **Unknown** | 3 |
| **N-stage** | **N0** | 65 (65.0) |
|  | **N1** | 17 (17.0) |
|  | **N2** | 14 (14.0) |
|  | **N3** | 4 (4.0) |
|  | **Unknown** | 3 |
| **TNM-stage** | **I** | 5 (5.0) |
|  | **II** | 24 (24.0) |
|  | **III** | 63 (63.0) |
|  | **IV** | 8 (8.0) |
|  | **Unknown** | 3 |
| **Histological grade** | **I** | 39 (40.2) |
|  | **II** | 40 (41.2) |
|  | **III** | 18 (18.6) |
|  | **Unknown** | 6 |

**Supplementary table 1.** Clinicopathological characteristic of the study population
